# Strain-engineered two-dimensional MoS_2_ as anode material for performance enhancement of Li/Na-ion batteries

**DOI:** 10.1038/s41598-018-20334-z

**Published:** 2018-02-01

**Authors:** Jiongyue Hao, Junfeng Zheng, Faling Ling, Yankun Chen, Huirong Jing, Tingwei Zhou, Liang Fang, Miao Zhou

**Affiliations:** 1 Key Laboratory of Optoelectronic Technology & Systems (Ministry of Education), College of Optoeletronic Engineering, Chongqing University, Chongqing, 400044 China; 2 Department of Applied Physics, College of Physics, Chongqing University, Chongqing, 401331 China

## Abstract

Recent years have witnessed a surge of research in two-dimensional (2D) nanostructures for development of new rechargeable Li/Na-ion battery systems. Herein, *via* first-principles calculations we demonstrate strain-engineered Li/Na adsorption and storage in 2D MoS_2_ as anode material, aiming to enhance the operating performance of Li/Na-ion batteries. Our results show that tensile strain greatly increases the adsorption of Li/Na atoms on MoS_2_, and a modest strain of 6% increases Li (Na) adsorption energy by over 70%, which originates from the strain-induced upshift of Mo *d* states towards Fermi level that interact strongly with Li/Na *s* states, in analogy with the *d*-band model in metal catalyst. Significant narrowing of the n-doped semiconducting gap of MoS_2_ suggests the improved electric conductivity that may benefit charge carrier transport. By mapping out the potential energy surfaces, we show shallow energy barriers of ion diffusion with ~0.2 eV for Li and 0.1 eV for Na. Furthermore, the strain-steered competition between chemical bonding and coulomb repulsion results in high Li/Na storage capability and relatively low average operating voltage. We believe that the fundamental principle underlying the use of strain to enhance performance of renewable ion battery is applicable to other stretchable low-dimensional nanomaterials.

## Introduction

With the rapid development of portable electronic devices, electric vehicles and implantable medical systems, rechargeable ion batteries with high energy and power densities have attracted great attention from both scientific and industrial communities. Among them, lithium ion batteries have been extensively studied and widely applied in consumer devices due to their advantages of compact size, high energy density and environmental friendliness^1,2^. However, the low natural abundance of Li reserves in together with uneven geographical spread limits large-scale electrical energy storage applications especially in the increasing electric vehicles and power grids^3,4^. In this regard, batteries using other metal ions such as Na have been considered to be promising alternatives for large-scale applications due to the abundant Na resources and low cost^5^. During the last decade, considerable efforts have been made to develop suitable electrolyte and electrode materials to enhance the operating performance of Li/Na batteries with high specific capacity, cyclic stability, high-rate capability and safety^6–10^.

Compared with cathode materials that may utilize a variety of structures, developing anode material seems to be more challenging. Graphite was used as the anode material for the first implementation of commercial Li ion batteries. However, it has a very low specific capacity (372 mAh/g) that cannot meet the requirements for large-scale electrical energy storage. For Na ion batteries, graphite is an inappropriate anode material due to the difficulty for Na intercalation into graphite. Metal oxides including TiO_2_, MnO_2_ and NiO have been proposed as promising anode materials with higher theoretical capacities than graphite^11–14^, but the poor electrical conductivity and structural stability during operating process lead to undesirable cycling performance. Thanks to the isolation of monolayer graphene, two-dimensional (2D) materials have currently become the focus of research due to their unique properties embracing high surface area, superior chemical stability tolerance and broad electrochemical window that hold great potential in energy technologies. As anode materials for ion batteries, previous studies have reported graphene nanosheets^15^, 2D transition metal dichalcogenides including MoS_2_, MoSe_2_ and NiSe_2_^16–18^, black phosphorene^19,20^, and the recently reported MXene^21–23^, borophene^24,25^, among many others^26–28^. These materials have different structures and exhibit diverse properties for the adsorption and diffusion of metal ions. In order to enhance the performance of ion batteries, great emphasis has been placed on tuning their electronic structures, i.e. via doping, decorating or making hybrid composites^29–31^. Therefore, to design an appropriate anode material and find an effective way to control its electronic property becomes a key issue for the future development of high-performance renewable batteries.

Strain engineering is one of the most common and generalizable methodologies to modulate the electronic structures of materials at an atomic level^32^. In semiconductor industry, strain effects on band structures of bulk Si is well understood^33,34^ and practically employed in practice to improve the mobility of charge carriers and switching speed of metal oxide semiconductor field-effect transistors. For 2D materials, elastic strain can be easily introduced on purpose or unintentionally, which offers an exciting opportunity to create strain-tunable optoelectronic and spintronic materials because they can sustain very high elastic strain before rupturing as compared to three-dimensional materials^35,36^. For instance, earlier reports have shown the possibility of strain-induced topological insulator state and pseudo-magnetic quantum Hall effect in graphene^37,38^. Band gap, effective mass and magnetism in phosphorene can also be effusively tuned by strain^39,40^. Recently, joint experimental and theoretical studies^41^ have shown an optimized catalytic activity of hydrogen evolution reaction on basal plane of MoS_2_ by straining the surface with S-vacancies, thus providing a new degree of freedom to manipulate the intrinsic activity of 2D nanocatalysts by strain.

In this work, we propose to use strain to engineer the adsorption, diffusion and storage capability of Li/Na ions on monolayer MoS_2_. Here, MoS_2_ is chosen as the typical anode material because in literature, it has been shown to exhibit excellent electrochemical performance for Li and Na storage^42^. Our systematic density functional theory (DFT) based first-principles calculations show that strain significantly increases the adsorption and storage of Li and Na. The electric conductivity of anode can also be improved due to the strain-induced narrowing of n-doped semiconducting gap of MoS_2_. Furthermore, we predicted low energy barriers of Li/Na ion diffusion and relatively low average operating voltages, which may contribute to the enhancement of operating performance. Remarkably, the underlying principle, especially the strain-induced upshift of Mo *d* states of MoS_2_ towards Fermi level that leads to stronger interaction with metal ions, is in close analogy with the *d*-band model in metal catalysts and can be generally applicable to other stretchable low-dimensional nanostructures. These results are expected to shed light on utilizing strain for future design of other energy storage systems.

## Computation and Details

**Figure 1:**
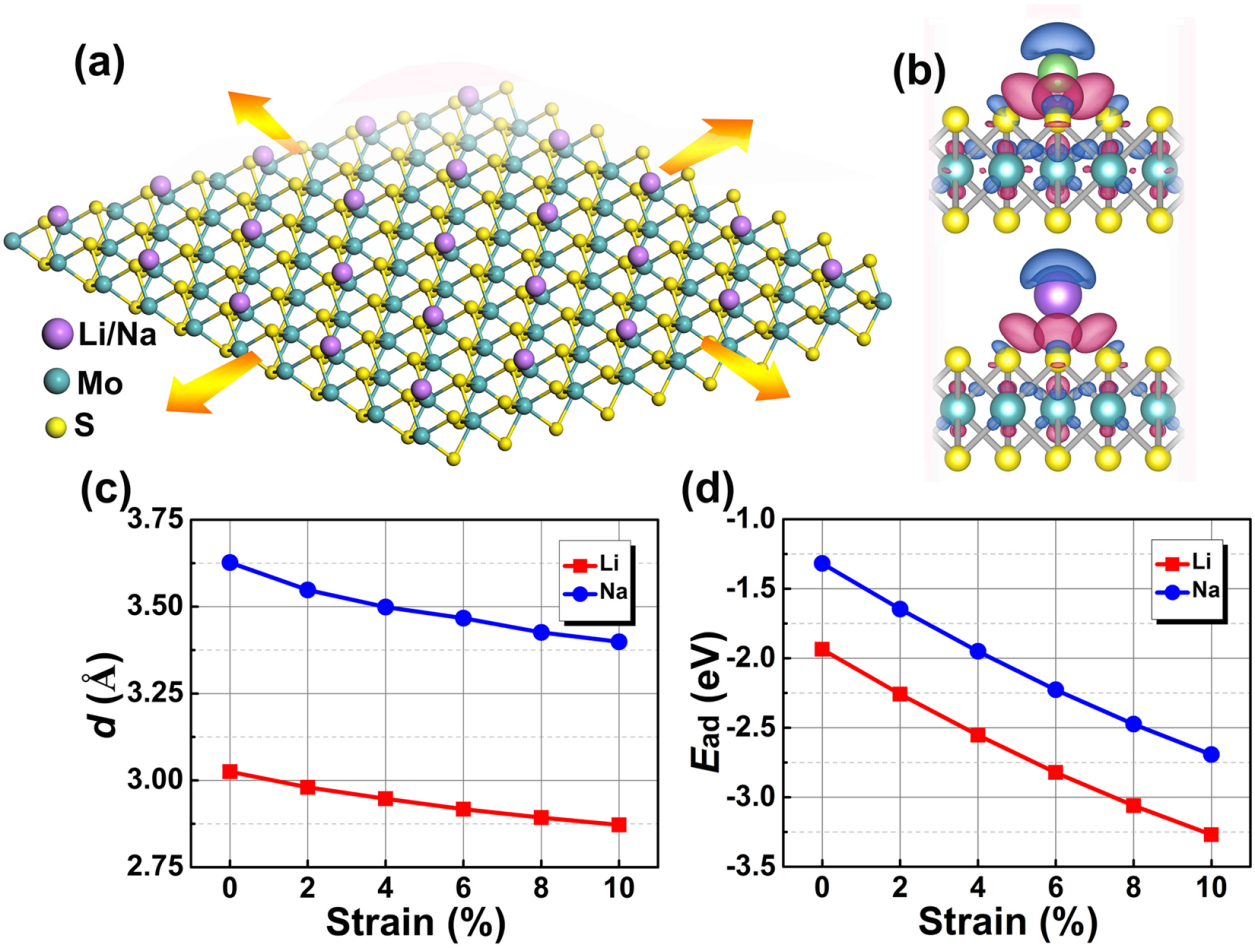
**(a)** A schematic view of Li/Na adsorbed on MoS_2_ sheet where tensile strain is uniformly applied. Purple, green and yellow balls represent Li/Na, Mo and S atoms, respectively. (**b**) The optimized most stable adsorption configuration for Li (upper panel) and Na (lower panel) atom on MoS_2_. Superimposed is the isosurface of differential charge density (isovalue = 0.02 *e*/Å^3^), which is calculated by $\,{\rm{\Delta }}\rho ={\rho }_{M@Mo{S}_{2}}-({\rho }_{M}+{\rho }_{Mo{S}_{2}})$, where $\,{\rho }_{M@Mo{S}_{2}}$, ${\rho }_{M}$ and ${\rho }_{Mo{S}_{2}}$ denote the charge density of the adsorbed system, Li/Na atom and MoS_2_, respectively. Charge depletion is in blue and accumulation in red. (**c**) The adsorption distance of Li (square) and Na (circle) against different magnitudes of tensile strain up to 10%. **(d)** The adsorption energy of Li and Na against strain.

Our DFT based first-principles calculations were carried out using the plane-wave-basis-set and the projector-augmented-wave (PAW) method^43,44^ as implemented in the VASP code^45^. The energy cutoff was set to 400 eV. For the exchange-correlation functional, we used the generalized gradient approximation (GGA) in Perdew-Burke-Ernzerhof (PBE) format^46,47^. We also checked GGA-PBE results by including van der Waals corrections (optPBE-vdW)^48,49^, which show no essential difference. Periodic images were separated by a vacuum region of over 20 Å in the direction perpendicular to the plane of MoS_2_ sheet to avoid spurious interaction between images. As a start, MoS_2_ with a 4 × 4 supercell was used to study the adsorption of Li/Na, and the Brillouin zone was sampled by a 12 × 12 × 1 *k*-grid. Denser *k*-point mesh was used for smaller supercells with a scaled fashion. For the density of states (DOS) calculations, Gaussian smearing with a smearing width of 0.1 eV has been used. Figure 1a shows a schematic view of Li/Na atoms adsorbed on monolayer MoS_2_, where tensile strain is uniformly applied. We considered different adsorption sites of Li/Na on the surface, including top site where metal atom sits directly above Mo, hollow site above the center of Mo-S hexagon, bridge site at the middle of Mo–S bond and the site directly above S atom. Structure relaxation is continued until forces on each atom were smaller than 0.01 eV/Å, after which the total energy and electronic structures were calculated and analyzed.

## Results and Discussion

As an anode material, it is crucial for the structure to attract Li/Na with relatively strong adsorption energy. Our calculations show that the most stable adsorption configuration with the lowest total energy for both Li and Na is the top site directly above Mo atom. In this configuration, the adsorption distance between Li (Na) and Mo atom is 3.02 Å (3.62 Å). This difference of distance is mainly due to the different ionic radii of Li and Na. To study the adsorption strength, we calculated the adsorption energy (${E}_{ad}$) defined as,

1 $${E}_{ad}={E}_{M@Mo{S}_{2}}-({E}_{M}+{E}_{Mo{S}_{2}})$$

where $\,{E}_{M@Mo{S}_{2}}$, ${E}_{M}$ and ${E}_{Mo{S}_{2}}\,$are energy of the total adsorbed system, of a single metal (M = Li/Na) atom and of the MoS_2_ layer, respectively. Within this definition, a more negative value of ${E}_{ad}$ suggests an energetically stronger adsorption (exothermic). Our calculated ${E}_{ad}$ for Li on MoS_2_ is −1.32 eV and for Na around −1.93 eV, in agreement with previous reports^42,50^. The nature of attraction between Li/Na and MoS_2_ can be analyzed by the differential charge density, as plotted in Fig. 1b. Clearly, significant charge transfer was observed from Li/Na to MoS_2_ surface and also the interface region. To quantitatively determine the charge transfer, we used the integration of differential charge density above the plane cutting through the middle point between Li/Na and MoS_2_ combined with Bader charge analysis method^51^. It was found that Li and Na lose 0.872 *e* and 0.853 *e* respectively, thus making them in the cationic state.

Then we apply tensile strain in MoS_2_, varying from 0% to 10% with an interval of 2%, and study its effects on Li/Na adsorption. Here, strain is defined as,

2 $${\rm{\varepsilon }}=(a-{a}_{0})/{a}_{0}$$

where ${a}_{0}$ and $a$ are the lattice constant of MoS_2_ without strain and with different strains, respectively. Figure 1c and d present the calculated adsorption difference and adsorption energy against strain. Interestingly, we can see that adsorption distance reduces greatly with increasing strain, from 3.02 Å (without strain) to less than 2.88 Å (with 10% of strain) for Li, and from 3.62 Å to 3.38 Å for Na. This is accompanied by significant increase of adsorption energy. As shown in Fig. 1d, the adsorption energy of Li increases from −1.93 eV without strain to −3.27 eV at 10% of strain, leading to an increase of nearly 70%. Similar effect was observed for Na, of which the adsorption energy increases from −1.32 eV to −2.69 eV, with an increase of over 100%. In particular, a modest strain of 6% increases Li (Na) adsorption by 46% (69%), which leads to much stronger adsorption compared to that without strain. Therefore, an applied tensile strain provides an effective approach to enhance Li/Na adsorption with high tunability.

**Figure 2:**
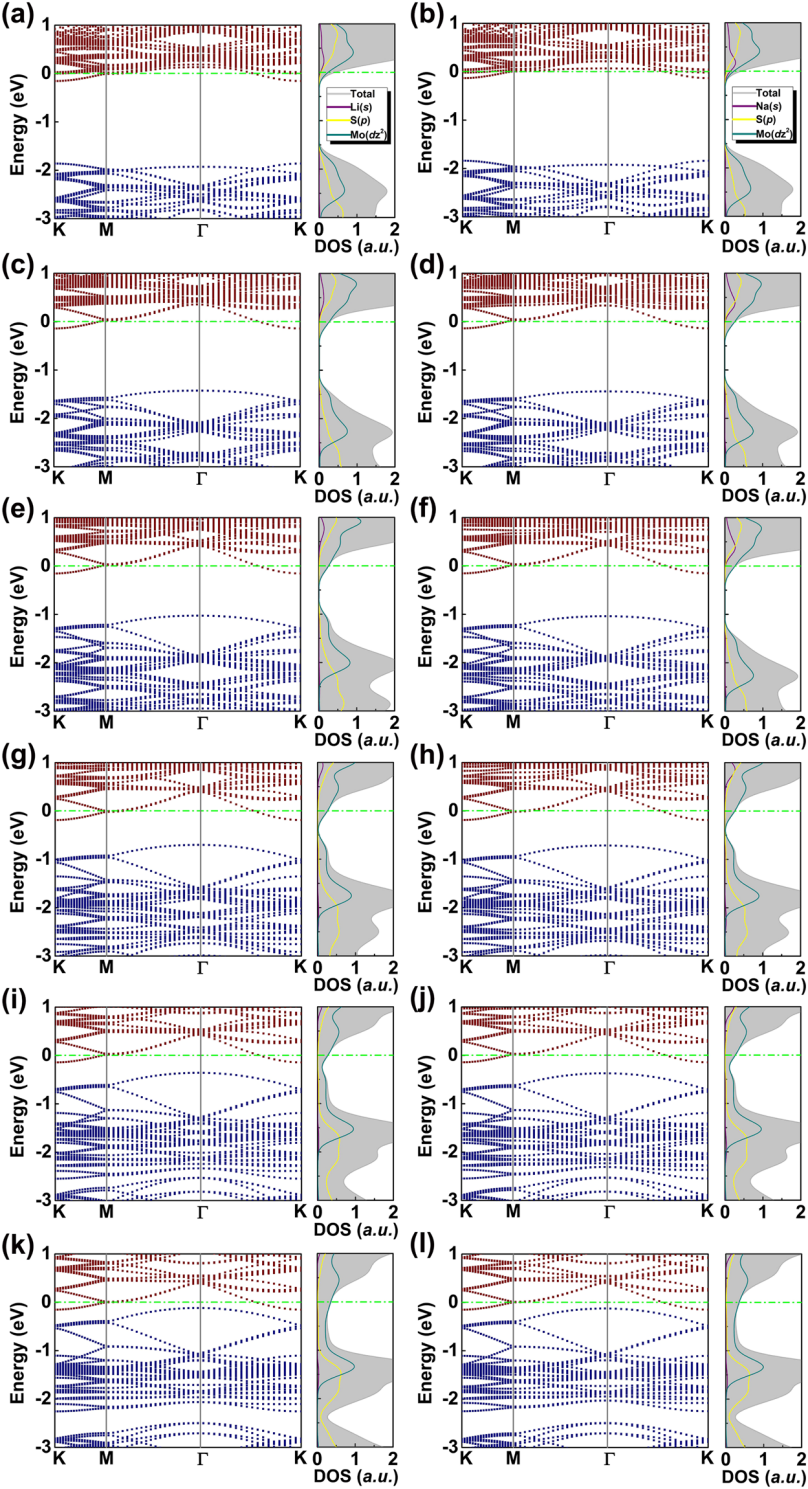
Band structures, total and partial DOS for Li and Na adsorption on MoS_2_ with different magnitudes of tensile strain. (**a**,**c**,**e**,**g i** and **k**) are for Li with 0%, 2%, 4%, 6%, 8% and 10% of strain, respectively. (**b,d**,**f**,**h**,**j** and **l**) are for Na. Fermi level is set to zero (green dashed line).

To study the strain effects on the electronic properties and understand the physical origin of the enhanced adsorption, we plot the band structures and DOS of the adsorbed structures. As shown in Fig. 2a and b, adsorption of Li (Na) shifts the Fermi level of pristine semiconducting MoS_2_ into the conduction band without strain, leaving MoS_2_ highly n-type doped. This is in accordance with charge transfer analysis, in which Li (Na) loses electrons and MoS_2_ gains electrons with an itinerant feature. Comparing Fig. 2a with Fig. 2c,e,g,i and k, we see that while Fermi level remains in the conduction band, the intrinsic band gap of MoS_2_ greatly reduces with increasing tensile strain as the valence band states moves up in energy towards Fermi level. Here, up-shift of valence bands with strain is closely related to their deformation potential, which has been intensively studied and well understood for conventional semiconductors, especially for the carrier mobility in Si. Here, with Li adsorption, the energy gap of MoS_2_ reduces from 1.86 eV without strain to 1.43 eV at 2% of strain, 1.03 eV at 4% of strain, 0.7 eV at 6% of strain and 0.36 eV at 8% of strain. Eventually, with 10% of tensile strain, the gap becomes negative as the conduction band minimum and valance band maximum overlaps with each other in the energy range (see Fig. 2k). Similar phenomena were also found for the adsorption of Na, as can be seen in Fig. 2b,d,f,h,j and l. This is particularly interesting because pristine MoS_2_ itself is a typical semiconductor that lacks superior electrical conductivity. By combining Li/Na adsorption with controllable strain engineering, we are able to manipulate the semiconducting gap and even achieve an unprecedented level to a metallic nature, which may be of a great benefit to charge carrier transport for potential applications in ion batteries and other electronic devices.

Figure 2 also includes the total and partial DOS of the adsorbed structures, from which we can learn more about the underlying atomic orbital hybridization between Li/Na and MoS_2_. Clearly, total DOS show reduction of the energy gap with increasing tensile strain, in accordance with the band structures. By projecting DOS to the *s* orbital of Li/Na, *p* orbitals of S and *d* orbitals of Mo, we found that *s* orbital of Li/Na overlaps with *p* orbitals of S and *d* orbitals of Mo in both conduction band and valence band energy ranges. Careful examination of the center of *d* orbitals of Mo suggests that it is the center of Mo *d* orbitals that shifts up in energy towards Fermi level with increased tensile strain, which contributes to the up-shift of valance bands. Remarkably, these shifted orbitals interact increasingly stronger with *s* orbital of Li/Na atoms. This reminds us of the *d*-band model in metal-based catalysts as proposed by Hammer and Nørskov^52^, which depicts a nice picture of using the location of *d*-band center as descriptor for the adsorption of small gas molecules on metal surface that greatly benefits efficient design of new catalysts^53^. Here, the center of *d* orbitals of Mo in MoS_2_ may also be used to predict the strength of *s*-*d* hybridization, i.e. Li/Na interaction with MoS_2_, and the strain tunable feature offers an additional advantage for the design of energy storage materials based on 2D structures.

The strain enhanced adsorption is also closely related to the charge transfer in the adsorbed systems. Our Bader charge analyses show that with increasing tensile strain, charge transfer from Li (Na) to MoS2 gradually increases, from 0.872 e (0.853 e) without strain to 0.876 e (0.868 e) at 10% of tensile, respectively, which makes Li (Na) more ‘cationic’ that increases the ionic bonding. These analyses are in good agreement with DOS calculations.

**Figure 3:**
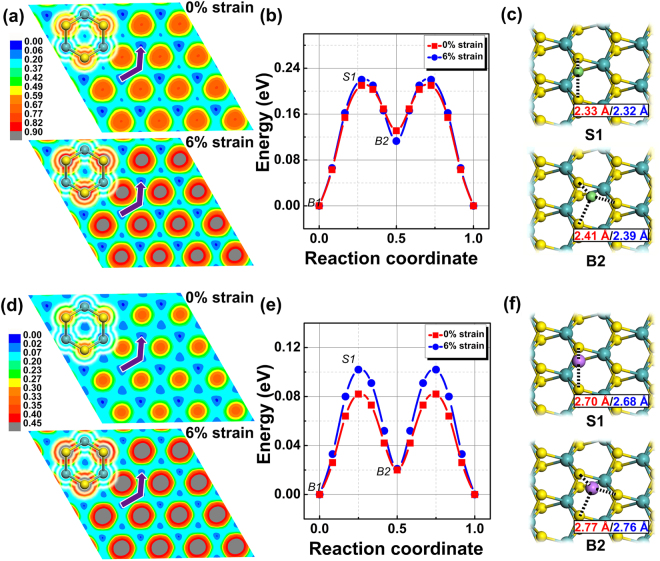
**(a)** PES of Li diffusion on MoS_2_ with 0% of strain and with 6% of strain. **(b)** Energy profile of Li diffusion along MEP as indicated by the arrow in **(a)**. B1 is the most stable top site, B2 is the metastable hollow site and S1 denotes the saddle point. **(c)** Local structure of S1 and B2, with the distance between Li and S indicated (red numbers for 0% of strain and blue for 6% of strain). **(d–f)** Cases for Na diffusion.

Mobility of ions on anode material plays a key role in determining the charging and circuit rate performance of batteries. It is desirable to investigate whether strain affects the diffusion of Li/Na on MoS_2_ surface. To quantify this, we calculated the potential energy surface (PES) of Li/Na diffusion on MoS_2_ without strain and with 6% of strain in order to precisely determine the minimum energy path (MEP) in together with the diffusion barriers. 6% of tensile strain is chosen as model in the following work, which is based on two reasons: 6% is a modest tensile strain that is readily achievable in real experiments^35,36^. Also, this magnitude of strain already has obvious effects on the adsorption energies of Li/Na atoms. Here, PES is calculated by fixing the adsorbed ions laterally at different positions and allowing all other atoms and the ion height to relax. In our study, we constructed 20 structures along zigzag and armchair directions of MoS_2_ and optimized a total of 400 structures to obtain the relative energy with reference to the lowest energy at the most stable adsorption site. Figure 3a and b show the PES and MEP for Li diffusion on MoS_2_, respectively. Without strain, we can see that Li migrates from the most stable top site (B1) to a metastable site (B2), which is above the center of Mo-S hexagon via a saddle point (S1), and then through a symmetric saddle point to the nearest most stable site. During this migration process, Li has to overcome a diffusion barrier of 0.21 eV. With 6% of strain, Li follows the same migration path with a very similar diffusion barrier of 0.22 eV. Figure 3c shows the local structures of Li at the saddle point S1 and the metastable site B2. It is found that Li bridges two S atoms at the saddle point, where the distance between Li and S remains almost unchanged (~2.32 Å) with strain. Therefore, tensile strain does not tend to affect the fast diffusion of Li on MoS_2_.

Figure 3d–f show the PES and MEP of Na migration on MoS_2_, where a similar diffusion path is observed. We found that Na has a much smaller diffusion barrier of 0.08 eV, suggesting much faster diffusion compared to Li on the surface. 6% of strain increases the diffusion barrier to around 0.1 eV, indicating little influence. The temperature-dependent diffusion rate can be evaluated by the Arrhenius equation, where the mean jump frequency (${\rm{\omega }}$) of Li/Na at temperature T can be expressed by ${\rm{\omega }}={{\rm{\upsilon }}e}^{(-{{\rm{E}}}_{b}/{{\rm{K}}}_{B}{\rm{T}})}.$ Here, the prefactor $\,{\rm{\upsilon }}$ relies on the lattice vibrations of diffusion states and diffusion barrier ${E}_{b}$ is the key to determine the ion kinetics. As strain does not affect the shallow energy barriers of ion diffusion significantly, i.e. with ~0.2 eV for Li and 0.1 eV for Na, we believe ions can readily diffuse during charging/discharging process at operating temperature.

**Figure 4:**
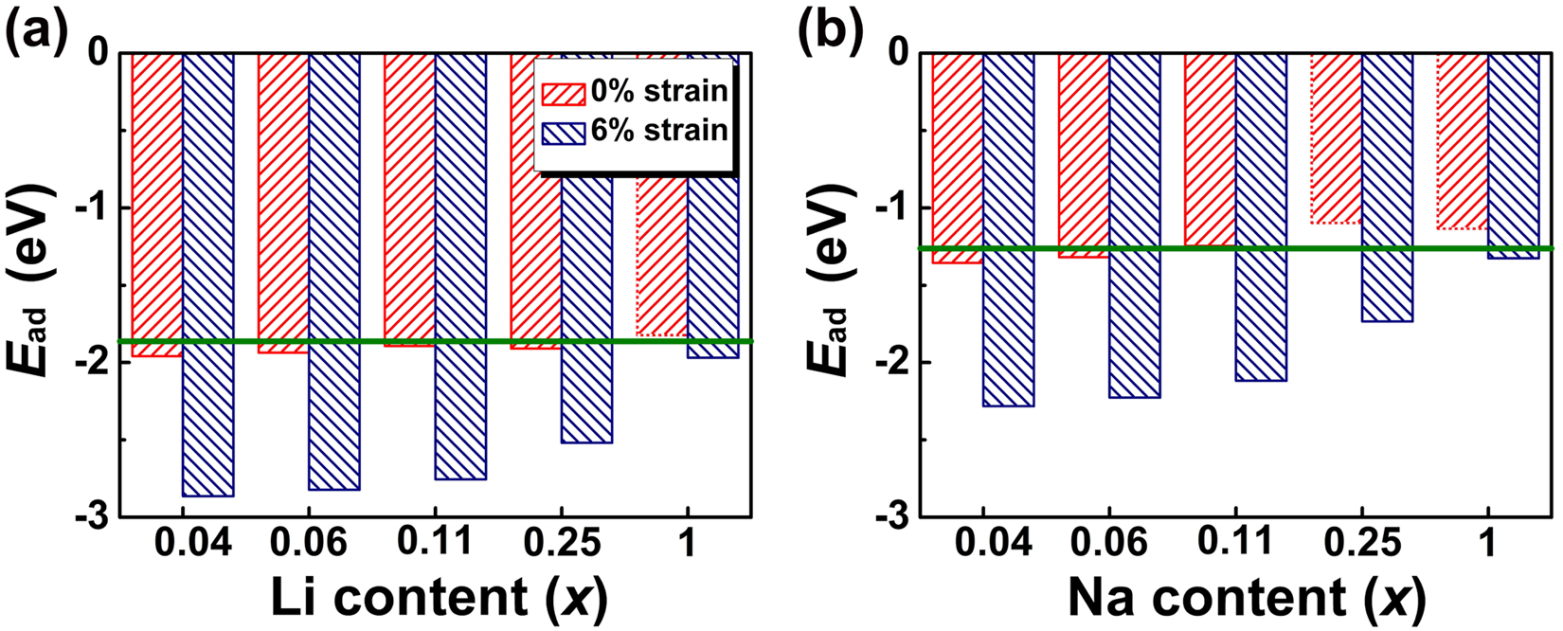
**(a)** Adsorption energy of Li on MoS_2_ under different concentrations with 0% of strain and with 6% of strain. Green line is chemical potential of bulk Li. **(b)** Same cases for Na adsorption, where the chemical potential of bulk Na is also indicated.

Theoretically, pristine MoS_2_ has a storage capacity of 670 mAh/g for Li batteries^54,55^, corresponding to one Li atom adsorption on every unit cell of MoS_2_. It was also predicted to have good rate capability and cycling stability due to the weak van der Waals interaction between MoS_2_ layers can facilitate Li intercalation and extraction without much volume change that prevents the pulverization problem^56^. However, in practice, significant capacity loss^57,58^ and continuous capacity fading during cycling (less than 200 cycles) were observed^59,60^, which was attributed to the very limited conductivity of MoS_2_ as we discussed above and the electrochemical degradation problem^60^. To get more physical insight into the degradation issue, we considered a series of adsorption concentrations with one Li/Na atom adsorbed on 1 × 1, 2 × 2, 3 × 3, 4 × 4 and 5 × 5 supercells of MoS_2_, corresponding to Li/Mo or Na/Mo stoichiometric ratios (*x*) of 1, 0.25, 0.11, 0.06 and 0.04, respectively. Our calculations show that with increasing adsorption concentration, adsorption energies of both Li and Na atom gradually decrease (Fig. 4). This is understandable because higher adsorption concentration leads to stronger coulomb repulsion between neighboring positively charged Li/Na ions. Specifically, with small stoichiometric ratios of 0.04 and 0.06, adsorption energies of Li are larger than its chemical potential of the bulk form, which was calculated to be −1.86 eV, so that Li can stay individually on the surface. With higher concentrations of 0.11 and 0.25, adsorption energies are comparable to the chemical potential, and with one Li atom adsorbed on a unit cell of MoS_2_, the adsorption energy becomes smaller than the chemical potential, suggesting that Li is not stable in the individual form and may cluster or form bulk Li. Therefore, instability of high-density storage may be the main driving force to the experimentally observed capacity loss and continuous capacity fading during cycling^61^.

Interestingly, with 6% of tensile strain, although the adsorption energy of Li decreases with increasing concentration, they are all much larger than its chemical potential (Fig. 4a), suggesting that the stability is significantly enhanced (even with the Li/Mo stoichiometric ratio of 1). For Na, the adsorption energy becomes smaller than its chemical potential (−1.26 eV) at the ratio of 0.11, indicating that the theoretical specific capacity is even harder to be reached as compared to Li. However, tensile strain turns to greatly increase the adsorption energies and stabilize the adsorption configuration (Fig. 4b). Therefore, our results show the possibility of manipulating the competition between chemical bonding (Li/Na and MoS_2_) and coulomb repulsion (between Li/Na ions) by strain, thus provide a promising approach to reduce the capacity loss/fading and achieve long lifetime of batteries.

Open-circuit-voltage is another important parameter that has been widely used for characterizing the performance of ion batteries, and has to be low for anode materials to get high voltage difference between cathode and anode of batteries. The open circuit voltage curve can be obtained by calculating the average voltage over parts of Li/Na composition domain^62,63^. The average voltage of the compound with stoichiometry with Li*x*MoS_2_ and Na*x*MoS_2_ in the range of ${x}_{1}$ ⩽ $x$ ⩽ ${x}_{2}$ can be expressed as,

3 $${\rm{V}}\approx \frac{{E}_{{M}_{x1}}-{E}_{{M}_{x2}}+({x}_{2}-{x}_{1}){E}_{M}}{({x}_{2}-{x}_{1})e}$$

where ${E}_{{M}_{x1}}$, ${E}_{{M}_{x2}}$, and ${E}_{M}$ are energies of Li ${x}_{1}\,$MoS_2_ (Na ${x}_{1}\,$MoS_2_), Li ${x}_{2}\,$MoS_2_ (Na ${x}_{2}\,$MoS_2_), and bulk Li (Na), respectively.

**Figure 5:**
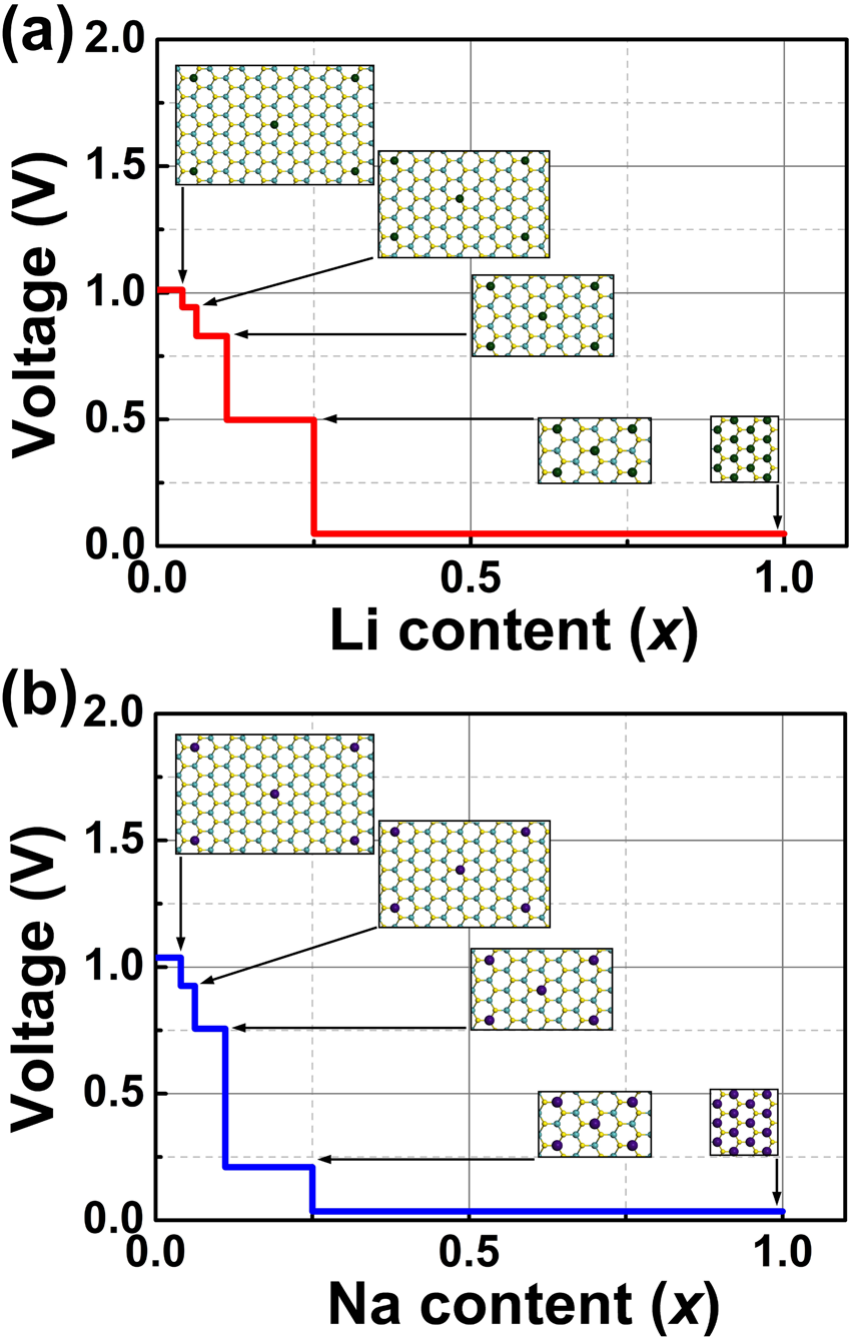
**(a)** The calculated voltage profile with respect to Li stoichiometric ratio from 0 to 1 with 6% of tensile strain. Insets show the configuration for each ratio. **(b)** The calculated voltage profile for Na.

We considered the aforementioned *x* = 0.04, 0.06, 0.11, 0.25 and 1 for Li*x*MoS_2_ and Na*x*MoS_2_, and the voltage profiles under 6% of strain associated with the configurations for each stoichiometry are presented in Fig. 5a and b, respectively. In general, voltage drops with increasing content of ions. At low Li concentration (0 ⩽ *x* ⩽ 0.06), relatively small voltage drops are observed. Dramatic drops can be seen when *x* > 0.06, with 0.33 V from *x* = 0.11 to 0.25 and 0.46 V from 0.25 to 1 (Fig. 5a). The calculated average voltage by averaging the whole voltage profile is 0.67 V. For Na, we found a rapid decrease from *x* = 0.11 to 0.25 with 0.55 V (Fig. 5b), and the average voltage was calculated to be 0.56 V. In terms of the low average open-circuit-voltage values, the strain tunable MoS_2_ could be a promising alternative for anode material.

## Conclusions

In summary, by using systematic first-principles calculations we explored the strain effects on the adsorption, diffusion and storage capability of Li/Na on MoS_2_ in the context of ion batteries. Our results demonstrate that tensile strain greatly increases the adsorption energy and narrows the n-doped semiconducting gap of MoS_2_, leading to enhanced stability with improved electrical conductivity. We highlight the *d* orbitals of Mo that can be effectively tuned by strain, which play a key role in determining the *s*-*d* hybridization and thus the Li/Na adsorption. Additionally, we demonstrate shallow energy barriers of ion diffusion that are not affected by strain, as well as the high storage capability and low average operating voltage, suggesting the promise of applying strain for energy related technologies.
